# Chromosome-Level Genome Assembly of the Asian Tramp Snail *Bradybaena similaris* (Stylommatophora: Camaenidae)

**DOI:** 10.1093/gbe/evaf070

**Published:** 2025-04-12

**Authors:** Yasuto Ishii, Atsushi Toyoda, Alec Lewis, Angus Davison, Osamu Miura, Kazuki Kimura, Satoshi Chiba

**Affiliations:** Graduate School of Life Sciences, Tohoku University, Sendai, Japan; Advanced Genomics Center, National Institute of Genetics, Mishima, Japan; School of Life Sciences, University of Nottingham, Nottingham, UK; School of Life Sciences, University of Nottingham, Nottingham, UK; Faculty of Agriculture and Marine Science, Department of Marine Resource Science, Marine Biological Chemistry Course, Kochi University, Kochi, Japan; Graduate School of Life Sciences, Tohoku University, Sendai, Japan; Center for Northeast Asian Studies, Tohoku University, Miyagi, Japan; Graduate School of Life Sciences, Tohoku University, Sendai, Japan; Center for Northeast Asian Studies, Tohoku University, Miyagi, Japan

**Keywords:** land snail, Mollusca, chromosome-scale assembly, Heterobranchia, Stylommatophora, Bradybaenidae

## Abstract

While terrestrial land snails have long been used for evolutionary research, a lack of high-quality genomic resources has impeded recent progress. *Bradybaena* snails in particular have numerous intriguing traits that make them a good model for studying evolution, including shell pattern polymorphism and convergent evolution. They are also introduced and invasive across the world. In this study, we present a chromosome-level genome assembly of the Asian tramp snail *Bradybaena similaris*, utilizing 88-fold Illumina short-read sequences, 125-fold Nanopore long-read sequences, 63-fold PacBio HiFi sequences, and 47-fold Hi-C sequences. The assembled genome of 2.18 Gb is anchored to 28 chromosomes and exhibits high completeness (single copy, 91.7%; duplicates, 7.1%) and contiguity (N50 of 75.6 Mb). Additionally, we also obtained a high-quality transcriptome for annotation. This resource represents the first chromosome-level assembly for snails in the superfamily Helicoidea, which includes more than 5,000 species of terrestrial snails, and will facilitate genomic study in *Bradybaena* and, more broadly, in the superfamily Helicoidea.

**Significance**

While the Helicoidea is the largest land snail superfamily, consisting of more than 5,000 species, many of interest to evolutionary studies, no chromosome-level assembly is available for any species. Previously, the genus *Bradybaena* in the Helicoidea has been studied in the context of speciation, adaptation, and invasive biology and thereby has high potential for further research. In this study, we present a chromosome-level assembly and transcriptome of the Asian tramp snail *Bradybaena similaris*. These high-quality genomic resources will facilitate research on related species and eventually enhance our understanding of many areas of evolutionary biology.

## Introduction

Terrestrial snails are relevant to many scientific fields. In the context of invasion biology, they are recognized as both agricultural and public health pests, leading to extensive research on their control (Hirano et al. 2020; Yonow et al. 2023). Unfortunately, in conservation biology, terrestrial snails are recognized as holding a position of significant risk (Chiba and Cowie 2016), representing the animal group with the most extinctions since the 16th century, with many species now requiring conservation efforts (Lydeard et al. 2004). Terrestrial snails also often serve as indicators of environmental pollution and so have been used in the study of anthropogenic impacts on the environment (Baroudi et al. 2020).

Terrestrial snails are a key group also in evolutionary biology (Chiba and Cowie 2016), especially species in the Helicoidea, the most speciose land snail superfamily, including more than 5,000 species (MolluscaBase 2024, accessed on 4th September 2024). For example, the genera *Satsuma* and *Euhadra* have been studied with the potential to understand speciation and also the origins of variation in left-right asymmetry (Ueshima and Asami 2003; Davison et al. 2005; Hoso et al. 2010; Richards et al. 2017). Also, research on *Cepaea*, *Theba*, and *Euhadra* continues to provide insight in understanding the evolution and genetics of shell color (Knigge et al. 2017; Ito et al. 2023; Johansen et al. 2023; Chowdhury et al. 2024). For another instance, in studies of behavioral evolution, *Cornu* and *Euhadra* have been major subjects of research on sexual conflict and mating behavior (Koene 2006; Kimura et al. 2013).

Despite these advantages as excellent model systems, genomic research has been scarce in terrestrial snails, partly due to a lack of high-quality genome assemblies (Davison and Neiman 2021; Linscott et al. 2022). As of now, genome assemblies are available for only eight land snail and slug species (supplementary table S1, Supplementary Material online). Among these, only the four genera, including *Achatina*, *Arion*, *Megaustenia*, and *Meghimatium*, have chromosome-level assemblies (Guo et al. 2019; Liu et al. 2021; Chen et al. 2022; Chetruengchai et al. 2024; Sun et al. 2024). Only two genome assemblies have been formally published for Helicoidea (*Candidula*: Chueca et al. 2021; *Cepaea*: Saenko et al. 2021), and no chromosome-level assembly has been obtained.

*Bradybaena* snails in the Helicoidea are particularly intriguing in the context of speciation and adaptation, where similar shell morphologies have evolved independently (Hirano et al. 2014, 2019). As shell morphology is a key trait for assortative mating (Kimura et al. 2015), then this may lead to parallel speciation. In the context of adaptation, the shell color and band pattern of *Bradybaena* snails are also important. *Bradybaena* snails have polymorphism in shell color and band pattern, with the variation likely maintained through balancing selection (Komai and Emura 1955). Each phenotype is determined by a single locus in the form of a supergene (Komai and Emura 1955), a concept that has recently received renewed attention (Berdan et al. 2022). Also, *Bradybaena* snails are also highly invasive around the world. *Bradybaena similaris*, native to East and Southeast Asia, has been introduced to every continent except Antarctica (Serniotti et al. 2019). This species damages not only crop but also hosts parasites, some of which can cause diseases in humans and livestock (Serniotti et al. 2019). Genomic resources could support further understanding of evolutionary innovations that make these invasive species and by facilitating the development of control strategies (North et al. 2021).

Here, we represent a chromosome-level genome assembly of *B. similaris*. Using high-coverage Nanopore long reads as well as PacBio HiFi reads, we successfully assembled a high-quality genome. Additionally, we obtained a high-quality transcriptome using newly sequenced RNA-seq data for annotation. The value in these genomic resources is their potential to facilitate research on *Bradybaena* snails, and more generally helicoidean snails, that can greatly benefit our understanding of multiple topics within evolutionary biology.

## Results and Discussion

**Fig. 1.**
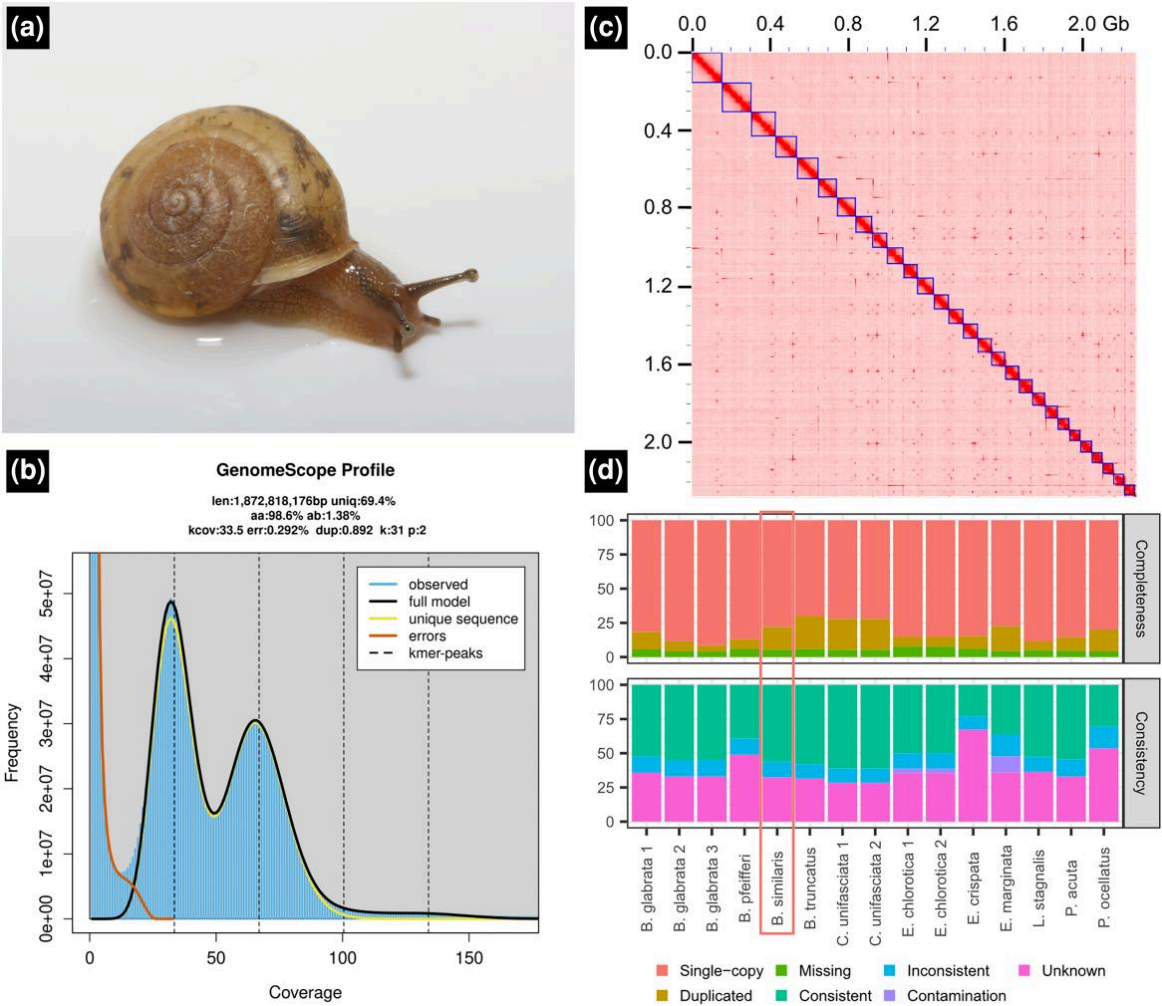
Overview of the results. a) Photograph of *B. similaris*. b) GenomeScope *k*-mer profile plot for *B. similaris*, displaying observed *k*-mer frequency (bar plot), full model fitted by GenomeScope (black line). len, estimated genome size; uniq, the proportion of non-repetitive sequences; aa, homozygosity rate; ab, heterozygosity rate; kcov, mean *k*-mer coverage for heterozygous bases; err, error rates of the reads; dup, average rate of read duplications; k, *k*-mer size; p, ploidy. c) Hi-C contact map for *B. similaris* with chromosomal scaffolds (blue boxes). Darker red indicates higher contact density. d) Comparison of OMArk statistics for Panpulmonata snails. All data were sourced from OMArk web server. The shown species and accession numbers (in parentheses) are the following: *Biomphalaria glabrata* (BglaB1, UP001165740, and GCF_947242115.1), *Biomphalaria pfeifferi* (GCA_030265305.1), *B. similaris* (this study), *Bulinus truncatus* (GCA_021962125.1), *C. unifasciata* (UP000678393 and GCA_905116865.2), *Elysia chlorotica* (UP000271974 and GCA_003991915.1), *Elysia crispate* (GCA_033675545.1), *Elysia marginata* (GCA_019649035.1), *Lymnaea stagnalis* (GCA_964033795.1), *Physella acuta* (GCF_028476545.1), and *Plakobranchus ocellatus* (GCA_019648995.1).

Sequencing yielded 192 Gb Illumina paired short-read sequences, 272 Gb Nanopore long-read sequences, 136 Gb PacBio HiFi sequences, and 101 Gb Hi-C library sequences (supplementary table S2, Supplementary Material online). Genome size and heterozygosity estimated by a *k*-mer-based method were 1,872,818,176 bp and 1.38%, respectively (Fig. 1b; Ranallo-Benavidez et al. 2020). An initial assembly generated by hifiasm (Cheng et al. 2022) had a size of 2,423,902,034 bp and consisted of 554 contigs (supplementary table S3, Supplementary Material online). After removing haplotigs, the assembly contained 279 contigs with the size of 2,289,349,004 bp and N50 of 17,829,327 bp (supplementary table S3, Supplementary Material online). Haplotig purging reduced the duplicated BUSCOs by about half (supplementary table S3, Supplementary Material online). This assembly was then scaffolded using Hi-C reads, producing a final assembly of 2,289,373,504 bp, comprising 82 scaffolds (Table 1). Some mis-joined chromosomal scaffolds were observed (supplementary fig. S1, Supplementary Material online), and they were split manually. Of these, 54 scaffolds (containing 270 contigs; 2,271,669,131 bp) were anchored onto 28 megascaffolds (Fig. 1c; supplementary table S4, Supplementary Material online), which corresponds to the haploid chromosome number of the species (Inaba 1959). The unanchored scaffolds are size of 17,704,373 bp and consisted of 57 contigs. Telomeric sequences were found in all but one of the chromosomal scaffolds; 15 and 12 chromosomal scaffolds had telomeric sequences on both sides and on one side, respectively (supplementary table S4, Supplementary Material online). The assembled genome size was larger than the genome size estimated using the *k*-mer method. This discrepancy has been observed in other large-genome organisms and is likely due to a high proportion of repeats (Pfenninger et al. 2022; Miura et al. 2023). The assembled genome has a higher contiguity and completeness than that of the other terrestrial snails (supplementary table S1, Supplementary Material online). The relatively high rate of BUSCO duplication (Table 1) should be a characteristic of land snails. The similar trend (duplication rate > 5%) is observed in land snail genomes, such as *Candidula*, 7.1%; *Cepaea*, 15.1%; *Achatina*, 6.9%; *Arion*, 6%; and *Oreohelix*, 9.1% (Guo et al. 2019; Chueca et al. 2021; Saenko et al. 2021; Chen et al. 2022; Linscott et al. 2022). The present study used metazoa_odb10 as a reference which is based on OrthoDB v10 (Kriventseva et al. 2019). OrthoDB v10 includes no stylommatophoran land snail genome (https://v10-1.orthodb.org/, accessed on 2025 March 29). Besides, stylommatophoran land snails experienced a whole-genome duplication event (Liu et al. 2021; Chen et al. 2022). Hence, the high rate of duplication could be explained by the taxon-specific duplication event. Future research should explore what core genes are duplicated in land snail genomes.

**Table 1:** The statistics for the genome assembly and annotation for *B. similaris*, after removing contaminants

Analysis	Statistics	Value
Assembly	Assembled genome size	2,289,373,504
	Number of contigs	327
	Number of scaffolds	82
	Contig N50 (Mb)	17.8
	Contig N90 (Mb)	5.0
	Scaffold N50 (Mb)	75.6
	Scaffold N90 (Mb)	56.1
	BUSCO completeness (%)	98.8
	Single copy (%)	91.7
	Duplicated (%)	7.1
	Fragmented (%)	0.3
	Missing (%)	0.9
Annotation	# of genes	29,226
	# of annotated genes	25,062
	Annotated using:	
	SwissProt	16,107
	TrEMBL	24,313
	EggNOG	22,863
	RefSeq invertebrate	23,452
	BUSCO completeness (%)	98.5
	Single copy (%)	91.8
	Duplicated (%)	6.7
	Fragmented (%)	0.4
	Missing (%)	1.1

The proportion of repeat content was estimated to be 61.28% (1.40 Gb), with long interspersed nuclear elements (LINEs) constituting a significant portion of the repeat content (19.51%; details are shown in supplementary table S5, Supplementary Material online). The proportion of LINEs is smaller than those of some terrestrial snails and slugs: *Candidula unifasciata*, 25.0% (Chueca et al. 2021); *Cepaea nemoralis*, 30.5% (Saenko et al. 2021); and *Meghimatium bilineatum*, 34.1% (Sun et al. 2024).

The genome was annotated using BRAKER3 (Gabriel et al. 2024), with 87.91 Gb RNA-seq data and OrthoDB 11 (Kuznetsov et al. 2023). Supplementary table S6, Supplementary Material online, shows the mapping rates for ten RNA-seq data used in this analysis. The number of predicted genes was 29,227. Functional annotation and contaminant detection were performed for the predicted genes using EnTAP (Hart et al. 2020). Any gene derived from bacteria was regarded as a putative contaminant. Using four protein sequence databases as a reference (SwissProt, TrEMBL, EggNOG, RefSeq invertebrate), a total of 25,062 genes were functionally annotated (Table 1). Among the annotated genes, 16,631 genes had Gene Ontology terms contained in EggNOG (Table 1). Only one gene was identified as a putative contaminant. Homology search was performed for this putative contaminant gene. The most homologous sequence was sourced from *Candidula* land snail (e-value = 3.16e-40), the second one was *Bulinus* freshwater snail (e-value = 1.97e-27), and the third one was *Physella* freshwater snail (e-value = 4.77e-26). Given this gene is found also in other molluscan genomes, we regarded that this gene is not a contaminant. The number of genes in the transcriptome is comparable to those of other land snails (supplementary table S5, Supplementary Material online).

Almost all metazoan core genes were found in the assembly and the transcriptome (98.8% and 98.5%, respectively; Table 1). The quality of the transcriptome was also assessed with OMArk (Nevers et al. 2024), which quantifies not only the completeness as BUSCO does (i.e. measuring how many expected genes are present in the genome) but also the consistency (i.e. the proportion of protein sequences correctly assigned to known gene families within the same lineage: Nevers et al. 2024). According to OMArk, 56.39% of the transcriptome was classified as “consistent,” 11.24% as “inconsistent,” and 32.37% as “unknown.” No gene was classified as “contaminant.” Although the relatively high proportion of inconsistent genes might suggest that the genome annotation can be improved, the quality was higher than that of most other Panpulmonata proteomes (i.e. higher “consistent” rate and lower “inconsistent” or “unknown” rate; Fig. 1d). The lack of model species in related taxa (Davison and Neiman 2021) may lead the relatively high proportion of inconsistent and unknown genes, as similar trends have been observed in other Panpulmonata proteomes (Fig. 1d).

## Conclusions

We presented a chromosome-level genome assembly for *B. similaris* utilizing Illumina paired-read sequences, Nanopore long-read sequences, HiFi long-read sequences, and Hi-C sequences. The assembled genome was 2.18 Gb in size, with N50 of 75.6 Mb and 98.8% metazoan BUSCO completeness (single copy, 91.7%; duplicates, 7.1%). This quality is much higher than that of the other published terrestrial snail genomes. The estimated transcriptome was also high-quality, containing 98.5% metazoan BUSCO (single copy, 91.8%; duplicates, 6.7%). These high-quality genome resources will accelerate the genomic study of terrestrial snails, particularly *Bradybaena* snails.

## Materials and Methods

### Sampling, DNA Extraction, and Sequencing

Three adult *B. similaris* individuals were collected for genome sequencing in Sendai, Miyagi, Japan (38.259661N 140.861679E) in September 2023 (Fig. 1a; supplementary table S2, Supplementary Material online). No permission is required to collect this species. All snail shells were pale-colored and lacked bands. These three individuals were separately used for short-read sequencing, long-read sequencing, and Hi-C sequencing (supplementary table S2, Supplementary Material online). DNA was extracted from a piece of foot using Nanobind Tissue Big DNA Kit (PacBio, US) for short-read sequencing and QIAGEN Genomic-tip 100/G (QIAGEN, Germany) for long-read sequencing.

An Illumina short-read library was prepared with a TruSeq DNA PCR-Free Library Prep Kit (Illumina, US). Two runs of 150 bp paired-end sequencing were then performed on a NovaSeq 6000 (Illumina) using the NovaSeq 6000 SP Reagent Kit v1.5 (Illumina). The Nanopore reads were obtained using a PromethION, a Ligation Sequencing Kit V14, and a R10.4.1 flowcell (Nanopore, UK). The PacBio library was prepared using a SMRTbell prep kit 3.0 and sequenced on both Sequel II and Sequel IIe platforms, using a Sequel II SMRT Cell 8 M along with a Sequel II Binding Kit 3.2 and Sequel II Sequencing Kit 2.0 (PacBio). These library preparations and sequencings were performed at the National Institute of Genetics Japan (NIG, Japan). An Hi-C library was prepared using Proximo Hi-C kit (Animal) following the manufacture's instruction. Sequencing was conducted on a DNBseq-g400 (MGI, China) at BGI.

RNA-seq was performed for annotation. An additional individual was collected at Sendai, Miyagi, Japan (38.259550N 140.850820E; sample ID: MNKS 6468). Tissue of the dart sac was preserved in RNAlater (QIAGEN) until extraction. Total RNA was extracted using ISOGEN (NIPPON GENE, Japan). Library preparation and sequencing were performed at BGI. DNBseq-g400 was utilized for 150 bp paired-end sequencing.

### De Novo Genome Assembly

De novo assembly was initially performed with Hifiasm 0.19.5-r587 (Cheng et al. 2022) using Nanopore as well as HiFi reads. Haplotigs were purged by purge_dups 1.2.5 (Guan et al. 2020), applying the “-e” option in the get_seqs module. The Hifiasm assembly was then used as input for Hi-C scaffolding. Hi-C reads were mapped onto the assembly using bwa 0.7.17-r1188 (Li, 2013) and samtools 1.17 (Danecek et al 2021). Then, the mapped reads were used for Hi-C scaffolding using YaHS 1.2 (Zhou et al. 2023). The contact map was visualized, and four pairs of scaffolds were clearly mis-joined into four chromosomes each, so these scaffolds were manually split. The final assembly was generated using Juicer 1.2 (Durand et al. 2016), Juicer tools 1.9.9, and JuiceBox 2.15.0.0 (Robinson et al. 2018).

Genome features, including genome size, heterozygosity, and repetitiveness, were estimated using GenomeScope 2.0 (Ranallo-Benavidez et al. 2020). The canonical *k*-mer distribution was produced from the short-read data with KMC 3.1.1 (Kokot et al. 2017) with *k*-mer length and maximum coverage set as 31 and 10,000, respectively.

### Genome Annotation

Prior to genome annotation, the repeat content was masked. RepeatModeler 2.0.4 (Flynn et al. 2020) was used to build a species-specific repeat content library. Using this library, the repeat content was masked using RepeatMasker 4.1.5 (http://www.repeatmasker.org). Both software tools were implemented in TETools 1.8 (https://github.com/Dfam-consortium/TETools).

Gene prediction was performed for the soft-masked sequence using BRAKER 3.0.8 (Gabriel et al. 2024). RNA-seq data (available at NCBI under accession numbers SRR8040510–SRR8040517, SRR6981555, and newly obtained data) and protein data (molluscan proteomes in OrthoDB 11; Kuznetsov et al. 2023) were used as reference data. The tissue for RNA-seq was sourced from whole body, embryo, digestive gland, and dart sac, among which dart sac data are newly obtained in this study. Functional annotation and contaminant detection for the predicted genes were carried out with EnTAP 1.3.0 (Hart et al. 2020). Four databases (SwissProt, TrEMBL, EggNOG, and RefSeq invertebrate) were referenced (O’Leary et al. 2016; Huerta-Cepas et al. 2019; UniProt Consortium 2023). Genes derived from bacteria were regarded as putative contaminants. Gene Ontology terms were assigned through EggNOG database. Homology search was performed for the putative contaminant transcriptome. blastp 2.14.0+ (Camacho et al. 2009) was adopted for this homology search against the NCBI nucleotide database.

Telomeric sequences on the final assembly were identified using the TeloExplorer module implemented in quarTeT 1.2.5 (Lin et al. 2023). An option, “-c animal,” was applied in this analysis. The gene content completeness of the assemblies and the transcriptome was assessed using BUSCO 5.7.1 (Manni et al. 2021), by searching against the metazoan core genes (metazoa_odb10). OMArk 0.3.0 (Nevers et al. 2024) was also used to quantify the quality of the annotation. To remove splicing variants, a transcript was extracted for each gene.

## Supplementary Material

evaf070_Supplementary_Data

## Data Availability

All raw data and assembly were deposited to DDBJ (BioProject accession number: PRJDB16720). All data are available from NCBI under the corresponding BioProject (verified on 2025 March 28). The accession numbers for each sequence are DRR528210 for Illumina short-read sequences, DRR623704–623707 for PacBio HiFi sequences, DRR527377 for Nanopore long-read sequences, DRR624568 for Hi-C sequences, and DRR633686 for RNA-seq sequences.

## References

[evaf070-B1] Baroudi F, Al Alam J, Fajloun Z, Millet M. Snail as sentinel organism for monitoring the environmental pollution; a review. Ecol Indic. 2020:113:106240. 10.1016/j.ecolind.2020.106240.

[evaf070-B2] Berdan EL, Flatt T, Kozak GM, Lotterhos KE, Wielstra B. Genomic architecture of supergenes: connecting form and function. Philos Trans R Soc Lond B Biol Sci. 2022:377(1856):20210192. 10.1098/rstb.2021.0192. 35694757 PMC9189501

[evaf070-B3] Camacho C, Coulouris G, Avagyan V, Ma N, Papadopoulos J, Bealer K, Madden TL. BLAST+: architecture and applications. BMC Bioinformatics. 2009:10(1):421. 10.1186/1471-2105-10-421. 20003500 PMC2803857

[evaf070-B4] Chen Z, Doğan Ö, Guiglielmoni N, Guichard A, Schrödl M. Pulmonate slug evolution is reflected in the de novo genome of *Arion vulgaris* Moquin-Tandon, 1855. Sci Rep. 2022:12(1):14226. 10.1038/s41598-022-18099-7. 35987814 PMC9392753

[evaf070-B5] Cheng H, Jarvis ED, Fedrigo O, Koepfli K-P, Urban L, Gemmell NJ, Li H. Haplotype-resolved assembly of diploid genomes without parental data. Nat Biotechnol. 2022:40(9):1332–1335. 10.1038/s41587-022-01261-x. 35332338 PMC9464699

[evaf070-B6] Chetruengchai W, Jirapatrasilp P, Srichomthong C, Assawapitaksakul A, Pholyotha A, Tongkerd P, Shotelersuk V, Panha S. De novo genome assembly and transcriptome sequencing in foot and mantle tissues of *Megaustenia siamensis* reveals components of adhesive substances. Sci Rep. 2024:14(1):13756. 10.1038/s41598-024-64425-6. 38877053 PMC11178922

[evaf070-B7] Chiba S, Cowie RH. Evolution and extinction of lands snails on oceanic islands. Annu Rev Ecol Evol Syst. 2016:47(1):123–141. 10.1146/annurev-ecolsys-112414-054331.

[evaf070-B8] Chowdhury M, Johansen M, Davison A. Continuous variation in the shell colour of the snail *Cepaea nemoralis* is associated with the colour locus of the supergene. J Evol Biol. 2024:37(9):1091–1100. 10.1093/jeb/voae093. 39066609

[evaf070-B9] Chueca LJ, Schell T, Pfenninger M. *De novo* genome assembly of the land snail *Candidula unifasciata* (Mollusca: Gastropoda). G3 (Bethesda). 2021:11(8):jkab180. 10.1093/g3journal/jkab180. 34849805 PMC8496239

[evaf070-B10] Danecek P, Bonfield JK, Liddle J, Marshall J, Ohan V, Pollard MO, Whitwham A, Keane T, McCarthy SA, Davies RM, et al Twelve years of SAMtools and BCFtools. Gigascience. 2021:10(2):1–4. 10.1093/gigascience/giab008. PMC7931819 33590861

[evaf070-B11] Davison A, Chiba S, Barton NH, Clarke B. Speciation and gene flow between snails of opposite chirality. PLoS Biol. 2005:3(9):e282. 10.1371/journal.pbio.0030282. 16149849 PMC1182688

[evaf070-B12] Davison A, Neiman M. Pearls of wisdom—a Theo Murphy issue on molluscan genomics. Philos Trans R Soc Lond B Biol Sci. 2021:376(1825):20200151. 10.1098/rstb.2020.0151. 33813890 PMC8059963

[evaf070-B13] Durand NC, Shamim MS, Machol I, Rao SSP, Huntley MH, Lander ES, Aiden EL. Juicer provides a one-click system for analyzing loop-resolution hi-C experiments. Cell Syst. 2016:3(1):95–98. 10.1016/j.cels.2016.07.002. 27467249 PMC5846465

[evaf070-B14] Flynn JM, Hubley R, Goubert C, Rosen J, Clark AG, Feschotte C, Smit AF. RepeatModeler2 for automated genomic discovery of transposable element families. Proc Natl Acad Sci U S A. 2020:117(17):9451–9457. 10.1073/pnas.1921046117. 32300014 PMC7196820

[evaf070-B15] Gabriel L, Brůna T, Hoff KJ, Ebel M, Lomsadze A, Borodovsky M, Stanke M. BRAKER3: fully automated genome annotation using RNA-seq and protein evidence with GeneMark-ETP, AUGUSTUS, and TSEBRA. Genome Res. 2024:34(5):769–777. 10.1101/gr.278090.123. 38866550 PMC11216308

[evaf070-B16] Guan D, McCarthy SA, Wood J, Howe K, Wang Y, Durbin R. Identifying and removing haplotypic duplication in primary genome assemblies. Bioinformatics. 2020:36(9):2896–2898. 10.1093/bioinformatics/btaa025. 31971576 PMC7203741

[evaf070-B17] Guo Y, Zhang Y, Liu Q, Huang Y, Mao G, Yue Z, Abe EM, Li J, Wu Z, Li S, et al A chromosomal-level genome assembly for the giant African snail *Achatina fulica*. Gigascience. 2019:8(10):1–8. 10.1093/gigascience/giz124. PMC6802634 31634388

[evaf070-B18] Hart AJ, Ginzburg S, Xu MS, Fisher CR, Rahmatpour N, Mitton JB, Paul R, Wegrzyn JL. EnTAP: bringing faster and smarter functional annotation to non-model eukaryotic transcriptomes. Mol Ecol Resour. 2020:20(2):591–604. 10.1111/1755-0998.13106. 31628884

[evaf070-B19] Hirano T, Kameda Y, Chiba S. Phylogeny of the land snails *Bradybaena* and *Phaeohelix* (Pulmonata: Bradybaenidae) in Japan. J Molluscan Stud. 2014:80(2):177–183. 10.1093/mollus/eyu004.

[evaf070-B20] Hirano T, Kameda Y, Saito T, Chiba S. Divergence before and after the isolation of islands: phylogeography of the *Bradybaena* land snails on the Ryukyu Islands of Japan. J Biogeogr. 2019:46(6):1197–1213. 10.1111/jbi.13575.

[evaf070-B21] Hirano T, Saito T, Shariar S, Tanchangya RTS, Chiba S. The first record of the introduced land snail *Bradybaena similaris* (Férussac, 1822) (Mollusca: Heterobranchia: Camaenidae) from Bangladesh. Bioinvasions Rec. 2020:9(4):730–736. 10.3391/bir.2020.9.4.07.

[evaf070-B22] Hoso M, Kameda Y, Wu S-P, Asami T, Kato M, Hori M. A speciation gene for left–right reversal in snails results in anti-predator adaptation. Nat Commun. 2010:1(1):1–7. 10.1038/ncomms1133. 21139578 PMC3105295

[evaf070-B23] Huerta-Cepas J, Szklarczyk D, Heller D, Hernández-Plaza A, Forslund SK, Cook H, Mende DR, Letunic I, Rattei T, Jensen LJ, et al eggNOG 5.0: a hierarchical, functionally and phylogenetically annotated orthology resource based on 5090 organisms and 2502 viruses. Nucleic Acids Res. 2019:47(D1):D309–D314. 10.1093/nar/gky1085. 30418610 PMC6324079

[evaf070-B24] Inaba A . Cytological studies in mollusks. II. A chromosome survey in the stylommatophoric Pulmonata. J Sci Hiroshima Univ Ser B Div. 1959:1(18):71–93.

[evaf070-B25] Ito S, Chiba S, Konuma J. Overcoming the congenitally disadvantageous mutation through adaptation to environmental UV exposure in land snails. Biol Lett. 2023:19(11):20230356. 10.1098/rsbl.2023.0356. 37990565 PMC10663782

[evaf070-B26] Johansen M, Saenko S, Schilthuizen M, Blaxter M, Davison A. Fine mapping of the *Cepaea nemoralis* shell colour and mid-banded loci using a high-density linkage map. Heredity (Edinb). 2023:131(5-6):327–337. 10.1038/s41437-023-00648-z. 37758900 PMC10673960

[evaf070-B27] Kimura K, Hirano T, Chiba S. Assortative mating with respect to size in the simultaneously hermaphroditic land snail *Bradybaena pellucida*. Acta Ethol. 2015:18(3):265–268. 10.1007/s10211-014-0211-7.

[evaf070-B28] Kimura K, Shibuya K, Chiba S. The mucus of a land snail love-dart suppresses subsequent matings in darted individuals. Anim Behav. 2013:85(3):631–635. 10.1016/j.anbehav.2012.12.026.

[evaf070-B29] Knigge T, Di Lellis MA, Monsinjon T, Köhler H-R. Relevance of body size and shell colouration for thermal absorption and heat loss in white garden snails, *Theba pisana* (Helicidae), from Northern France. J Therm Biol. 2017:69:54–63. 10.1016/j.jtherbio.2017.06.001. 29037405

[evaf070-B30] Koene JM . Tales of two snails: sexual selection and sexual conflict in *Lymnaea stagnalis* and *Helix aspersa*. Integr Comp Biol. 2006:46(4):419–429. 10.1093/icb/icj040. 21672754

[evaf070-B31] Kokot M, Dlugosz M, Deorowicz S. KMC 3: counting and manipulating k-mer statistics. Bioinformatics. 2017:33(17):2759–2761. 10.1093/bioinformatics/btx304. 28472236

[evaf070-B32] Komai T, Emura S. A study of population genetics on the polymorphic land snail *Bradybaena similaris*. Evolution. 1955:9(4):400–418. 10.1111/j.1558-5646.1955.tb01550.x.

[evaf070-B33] Kriventseva EV, Kuznetsov D, Tegenfeldt F, Manni M, Dias R, Simão FA, Zdobnov EM. OrthoDB v10: sampling the diversity of animal, plant, fungal, protist, bacterial and viral genomes for evolutionary and functional annotations of orthologs. Nucleic Acids Res. 2019:47(D1):D807–D811. 10.1093/nar/gky1053. 30395283 PMC6323947

[evaf070-B34] Kuznetsov D, Tegenfeldt F, Manni M, Seppey M, Berkeley M, Kriventseva EV, Zdobnov EM. OrthoDB v11: annotation of orthologs in the widest sampling of organismal diversity. Nucleic Acids Res. 2023:51(D1):D445–D451. 10.1093/nar/gkac998. 36350662 PMC9825584

[evaf070-B35] Li H. 2013. Aligning sequence reads, clone sequences and assembly contigs with BWA-MEM. arXiv:1303.3997 [q-bio.GN], preprint: not peer reviewed. 10.48550/ARXIV.1303.3997

[evaf070-B36] Lin Y, Ye C, Li X, Chen Q, Wu Y, Zhang F, Pan R, Zhang S, Chen S, Wang X, et al Quartet: a telomere-to-telomere toolkit for gap-free genome assembly and centromeric repeat identification. Hortic Res. 2023:10(8):uhad127. 10.1093/hr/uhad127. 37560017 PMC10407605

[evaf070-B37] Linscott TM, González-González A, Hirano T, Parent CE. De novo genome assembly and genome skims reveal LTRs dominate the genome of a limestone endemic Mountainsnail (*Oreohelix idahoensis*). BMC Genomics. 2022:23(1):796. 10.1186/s12864-022-09000-x. 36460988 PMC9719178

[evaf070-B38] Liu C, Ren Y, Li Z, Hu Q, Yin L, Wang H, Qiao X, Zhang Y, Xing L, Xi Y, et al Giant African snail genomes provide insights into molluscan whole-genome duplication and aquatic-terrestrial transition. Mol Ecol Resour. 2021:21(2):478–494. 10.1111/1755-0998.13261. 33000522

[evaf070-B39] Lydeard C, Cowie RH, Ponder WF, Bogan AE, Bouchet P, Clark SA, Cummings KS, Frest TJ, Gargominy O, Herbert DG, et al The global decline of nonmarine mollusks. Bioscience. 2004:54(4):321. 10.1641/0006-3568(2004)054[0321:tgdonm]2.0.co;2.

[evaf070-B40] Manni M, Berkeley MR, Seppey M, Simão FA, Zdobnov EM. BUSCO update: novel and streamlined workflows along with broader and deeper phylogenetic coverage for scoring of eukaryotic, prokaryotic, and viral genomes. Mol Biol Evol. 2021:38(10):4647–4654. 10.1093/molbev/msab199. 34320186 PMC8476166

[evaf070-B41] Miura O, Toyoda A, Sakurai T. Chromosome-scale genome assembly of the freshwater snail *Semisulcospira habei* from the Lake Biwa drainage system. Genome Biol Evol. 2023:15(11):evad208. 10.1093/gbe/evad208. 38014863 PMC10683039

[evaf070-B42] MolluscaBase eds. (2024). MolluscaBase. [accessed 2024 Sep 4]. https://www.molluscabase.org.

[evaf070-B43] Nevers Y, Warwick Vesztrocy A, Rossier V, Train C-M, Altenhoff A, Dessimoz C, Glover NM. Quality assessment of gene repertoire annotations with OMArk. Nat Biotechnol. 2024:43(1):124–133. 10.1038/s41587-024-02147-w. 38383603 PMC11738984

[evaf070-B44] North HL, McGaughran A, Jiggins CD. Insights into invasive species from whole-genome resequencing. Mol Ecol. 2021:30(23):6289–6308. 10.1111/mec.15999. 34041794

[evaf070-B45] O’Leary NA, Wright MW, Brister JR, Ciufo S, Haddad D, McVeigh R, Rajput B, Robbertse B, Smith-White B, Ako-Adjei D, et al Reference sequence (RefSeq) database at NCBI: current status, taxonomic expansion, and functional annotation. Nucleic Acids Res. 2016:44(D1):D733–D745. 10.1093/nar/gkv1189. 26553804 PMC4702849

[evaf070-B46] Pfenninger M, Schönnenbeck P, Schell T. ModEst: accurate estimation of genome size from next generation sequencing data. Mol Ecol Resour. 2022:22(4):1454–1464. 10.1111/1755-0998.13570. 34882987

[evaf070-B47] Ranallo-Benavidez TR, Jaron KS, Schatz MC. GenomeScope 2.0 and Smudgeplot for reference-free profiling of polyploid genomes. Nat Commun. 2020:11(1):1432. 10.1038/s41467-020-14998-3. 32188846 PMC7080791

[evaf070-B48] Richards PM, Morii Y, Kimura K, Hirano T, Chiba S, Davison A. Single-gene speciation: mating and gene flow between mirror-image snails. Evol Lett. 2017:1(6):282–291. 10.1002/evl3.31. 30283656 PMC6121799

[evaf070-B49] Robinson JT, Turner D, Durand NC, Thorvaldsdóttir H, Mesirov JP, Aiden EL. Juicebox.Js provides a cloud-based visualization system for hi-C data. Cell Syst. 2018:6(2):256–258.e1. 10.1016/j.cels.2018.01.001. 29428417 PMC6047755

[evaf070-B50] Saenko SV, Groenenberg DSJ, Davison A, Schilthuizen M. The draft genome sequence of the grove snail *Cepaea nemoralis*. G3 (Bethesda). 2021:11(2):jkaa071. 10.1093/g3journal/jkaa071. 33604668 PMC8022989

[evaf070-B51] Serniotti EN, Guzmán LB, Beltramino AA, Vogler RE, Rumi A, Peso JG. New distributional records of the exotic land snail *Bradybaena similaris* (Férussac, 1822) (Gastropoda, Bradybaenidae) in Argentina. Bioinvasions Rec. 2019:8(2):301–313. 10.3391/bir.2019.8.2.12.

[evaf070-B52] Sun S, Han X, Han Z, Liu Q. Chromosomal-scale genome assembly and annotation of the land slug (*Meghimatium bilineatum*). Sci Data. 2024:11(1):35. 10.1038/s41597-023-02893-7. 38182611 PMC10770140

[evaf070-B53] Ueshima R, Asami T. Evolution: single-gene speciation by left-right reversal. Nature. 2003:425(6959):679. 10.1038/425679a. 14562091

[evaf070-B54] UniProt Consortium . UniProt: the universal protein knowledgebase in 2023. Nucleic Acids Res. 2023:51(D1):D523–D531. 10.1093/nar/gkac1052. 36408920 PMC9825514

[evaf070-B55] Yonow T, Kriticos DJ, Zalucki MP, McDonnell JR, Caron V. Population modelling for pest management: a case study using a pest land snail and its fly parasitoid in Australia. Ecol Modell. 2023:482:110413. 10.1016/j.ecolmodel.2023.110413.

[evaf070-B56] Zhou C, McCarthy SA, Durbin R. YaHS: yet another Hi-C scaffolding tool. Bioinformatics. 2023:39(1):btac808. 10.1093/bioinformatics/btac808. 36525368 PMC9848053

