# Supplementary material for: Chromosome-Level Genome Assembly of the Asian Tramp Snail Bradybaena similaris (Stylommatophora: Camaenidae)
Source: Genome Biol Evol. 2025 Apr 12;17(5):evaf070. doi: 10.1093/gbe/evaf070 (PMC12042801; doi:10.1093/gbe/evaf070)
Supplement: evaf070_Supplementary_Data [file evaf070_supplementary_data.docx]

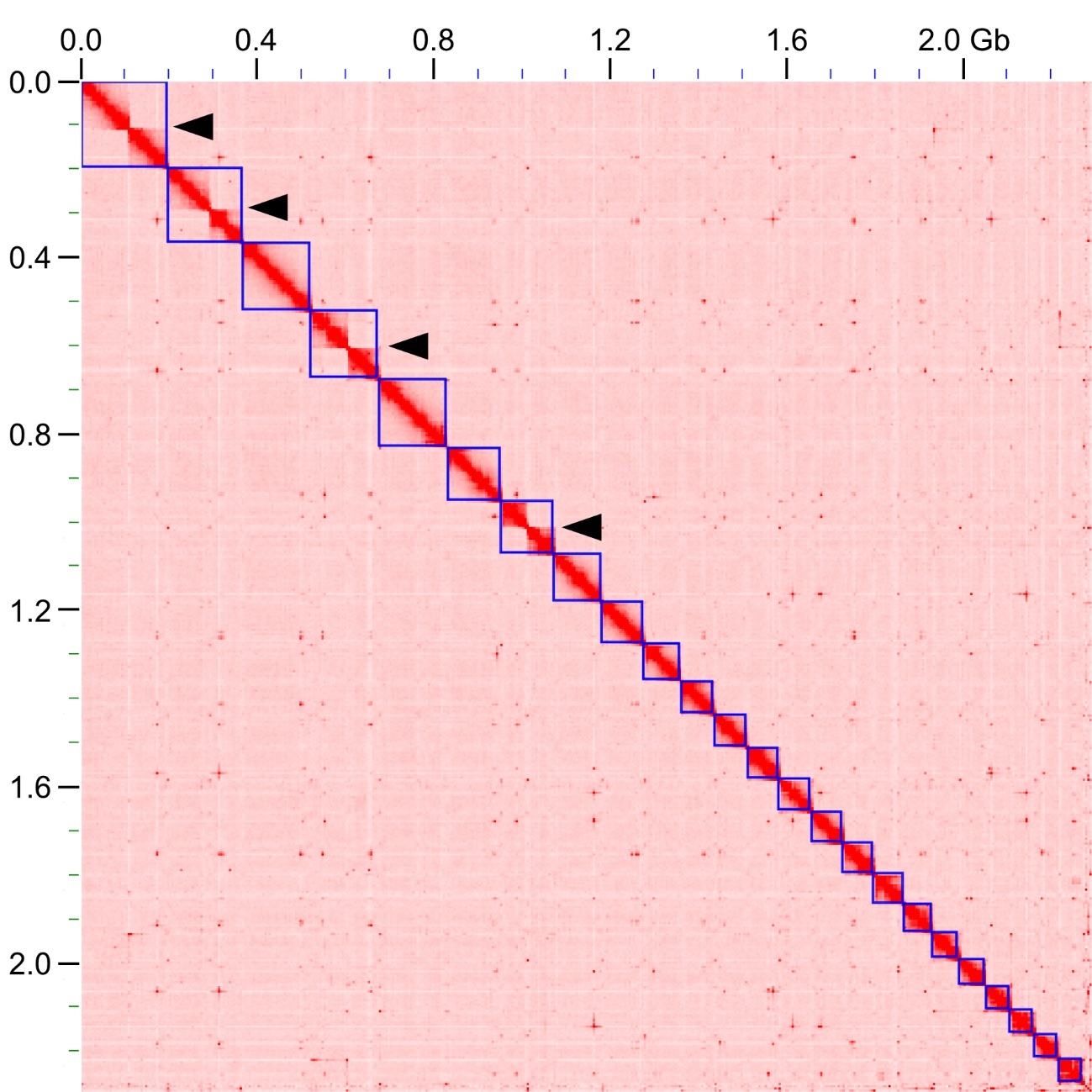


Fig. S1. Hi-C contact map before manual editing for *Bradybaena similaris*. Blue boxes indicate chromosomal scaffolds. Black triangles point mis-join chromosomal scaffolds. Darker red indicates higher contact density.

Table S1. Comparison of published assemblies of land snails. The complete BUSCO values are obtained for the genome sequences. NA: not available.

| **Species** | **Assembly size [Gb]** | **Contig N50 [Kb]** | **#Contigs** | **Scaffold N50 [Mb]** | **#Scaffolds** | **Complete BUSCO** | **#Genes** | **Reference** |
| --- | --- | --- | --- | --- | --- | --- | --- | --- |
| *Bradybaena similaris* | 2.29 | 17,829 | 279 | 75.6 | 82 | 98.8 | 29,226 | The present study |
| *Megaustenia siamensis* | 2.6 | 1,500 | 5,246 | 84.3 | 160 | 85.9 | 34,882 | Chetruengchai et al. (2024) |
| *Meghimatium bilineatum* | 1.5 | 1,370 | 2,526 | 68.08 | 2,528 | 91.7 | 11,816 | Sun et al. (2024) |
| *Arion vulgaris* | 1.54 | 8,600 | 7,076 | 63.3 | 6,751 | 90.6 | 32,518 | Chen et al. (2022) |
| *Candidula unifasciata* | 1.29 | 205 | 11,756 | 0.246 | 8,586 | 92.4 | 22,464 | Chueca et al. (2021) |
| *Oreohelix idahoensis* | 5.40 | - | - | 0.404 | 23,228 | 86.6 | 27,692 | Linscott et al. (2022) |
| *Achatina immaculata* | 1.75 | 3,800 | 563 | - | - | 93.2 | 30,194 | Liu et al. (2021) |
| *Cepaea nemoralis* | 3.49 | 330 | 28,537 | - | - | 89.0 | 43,519 | Saenko et al. (2021) |
| *Achatina fulica* | 2.12 | 721 | 8,211 | 59.5 | 1,010 | 91.7 | 23,726 | Guo et al. (2019) |

Chen Z, Doğan Ö, Guiglielmoni N, Guichard A, Schrödl M. 2022. Pulmonate slug evolution is reflected in the de novo genome of *Arion vulgaris* Moquin-Tandon, 1855. Sci Rep. 12:14226. https://doi.org/10.1038/s41598-022-18099-7

Chetruengchai W et al. 2024. De novo genome assembly and transcriptome sequencing in foot and mantle tissues of *Megaustenia siamensis* reveals components of adhesive substances. Sci Rep. 14:13756. https://doi.org/10.1038/s41598-024-64425-6

Chueca LJ, Schell T, Pfenninger M. 2021. *De novo* genome assembly of the land snail *Candidula unifasciata* (Mollusca: Gastropoda). G3. 11:jkab180. https://doi.org/10.1093/g3journal/jkab180

Guo Y et al. 2019. A chromosomal-level genome assembly for the giant African snail *Achatina fulica*. Gigascience. 8:1–8. https://doi.org/10.1093/gigascience/giz124

Linscott TM, González-González A, Hirano T, Parent CE. 2022. De novo genome assembly and genome skims reveal LTRs dominate the genome of a limestone endemic Mountainsnail (*Oreohelix idahoensis*). BMC Genomics. 23:796. https://doi.org/10.1186/s12864-022-09000-x

Liu C et al. 2021. Giant African snail genomes provide insights into molluscan whole-genome duplication and aquatic-terrestrial transition. Mol Ecol Resour. 21:478–494. https://doi.org/10.1111/1755-0998.13261

Saenko SV, Groenenberg DSJ, Davison A, Schilthuizen M. 2021. The draft genome sequence of the grove snail *Cepaea nemoralis*. G3. 11: jkaa071. https://doi.org/10.1093/g3journal/jkaa071

Sun S, Han X, Han Z, Liu Q. 2024. Chromosomal-scale genome assembly and annotation of the land slug (*Meghimatium bilineatum*). Sci Data. 11:35. https://doi.org/10.1038/s41597-023-02893-7

Table S2. Sequencing results. Different ID indicates a distinct individual.

| **Collection ID** | **Library type** | **Sequencer** | **#base** |
| --- | --- | --- | --- |
| YIPCMo-001276 | Short-read | Novaseq 6000 (Illumina) | 192,609,120,300 |
| YIPCMo-001421 | Long-read | PromethION (Nanopore) | 272,404,171,079 |
| YIPCMo-001421 | HiFi | Sequel II, Sequel IIe (PacBio) | 136,667,671,959 |
| YIPCMo-001540 | Hi-C | DNBseq-g400 (MGI) | 101,897,429,100 |

Table S3. Statistics for the intermediate assemblies. The four values in the BUSCO column indicate the proportion of single-copy, duplicated, fragmented, and missing BUSCO, respectively.

| **Mode** | **Size [bp]** | **#Contig** | **Contig N50 [Mb]** | **Contig N90 [Mb]** | **BUSCO [%]** |
| --- | --- | --- | --- | --- | --- |
| hifiasm | 2,423,902,034 | 554 | 17.2 | 3.5 | 88.1 / 9.5 / 1.2 / 1.2 |
| Hifiasm + Purge_Dups | 2,289,349,004 | 279 | 17.8 | 5.0 | 91.1 / 6.4 / 1.2 / 1.3 |

Table S4. Features of chromosomal scaffolds. In the "#Telomeric repeat" column, the first and second numbers indicate the number of telomeric repeats at the beginning and end of each scaffold, respectively.

| **Scaffold ID** | **Size [bp]** | **#Telomeric repeat** |
| --- | --- | --- |
| Scaffold 1 | 156,962,129 | 2177 / 705 |
| Scaffold 2 | 152,881,578 | 2631 / 1779 |
| Scaffold 3 | 123,925,688 | 2518 / 1468 |
| Scaffold 4 | 109,445,529 | 0 / 0 |
| Scaffold 5 | 107,517,165 | 3580 / 1907 |
| Scaffold 6 | 96,078,044 | 2915 / 0 |
| Scaffold 7 | 95,339,905 | 0 / 1058 |
| Scaffold 8 | 86,895,567 | 0 / 1991 |
| Scaffold 9 | 82,375,257 | 1898 / 2681 |
| Scaffold 10 | 81,539,857 | 2800 / 3113 |
| Scaffold 11 | 75,675,324 | 0 / 3076 |
| Scaffold 12 | 74,815,354 | 1989 / 249 |
| Scaffold 13 | 74,575,081 | 3102 / 0 |
| Scaffold 14 | 73,869,365 | 4298 / 0 |
| Scaffold 15 | 73,183,519 | 0 / 2519 |
| Scaffold 16 | 73,088,726 | 1700 / 0 |
| Scaffold 17 | 71,692,360 | 1860 / 1813 |
| Scaffold 18 | 69,625,931 | 1952 / 673 |
| Scaffold 19 | 66,548,126 | 2156 / 0 |
| Scaffold 20 | 65,701,203 | 2759 / 0 |
| Scaffold 21 | 63,384,139 | 2102 / 3725 |
| Scaffold 22 | 60,798,888 | 2396 / 3199 |
| Scaffold 23 | 58,091,544 | 2685 / 0 |
| Scaffold 24 | 57,229,777 | 994 / 2597 |
| Scaffold 25 | 56,138,484 | 874 / 1657 |
| Scaffold 26 | 55,777,177 | 3040 / 1526 |
| Scaffold 27 | 55,296,711 | 847 / 717 |
| Scaffold 28 | 53,216,703 | 0 / 6200 |

Table S5. The result for repeat content analysis.

| **Repeat element** | **Count** | **Length** | **Proportion [%]** |
| --- | --- | --- | --- |
| Retroelements | 1,540,365 | 517,662,341 | 22.61 |
| SINEs | 38,174 | 5,746,394 | 0.25 |
| LINEs | 1,443,421 | 446,737,655 | 19.51 |
| LTR | 58,770 | 65,178,292 | 2.85 |
| Penelope | 0 | 0 | 0.00 |
| DNA transposons | 430,140 | 146,285,966 | 6.39 |
| Simple repeats | 981,396 | 100,108,658 | 4.37 |
| Satellite | 13,306 | 3,142,698 | 0.14 |
| Low complexity | 64,914 | 6,203,086 | 0.27 |
| Unclassified | 3,257,079 | 623,596,281 | 27.24 |

Table S6. Mapping rate of RNA-seq data. The asterisk indicates the sample sequenced in the present study.

| **Accession number** | **Tissue** | **Mapping rate [%]** |
| --- | --- | --- |
| *DRR633686 | Dart sac | 52.33 |
| SRR6981555 | Whole body | 20.07 |
| SRR8040510 | Embryo | 52.64 |
| SRR8040511 | Embryo | 50.03 |
| SRR8040512 | Embryo | 54.89 |
| SRR8040513 | Embryo | 53.45 |
| SRR8040514 | Digestive gland | 69.60 |
| SRR8040515 | Digestive gland | 68.26 |
| SRR8040516 | Whole body | 70.75 |
| SRR8040517 | Whole body | 55.04 |
